# Thoracic high resolution computed tomography evaluation of imaging abnormalities of 108 lung cancer patients with different pulmonary function

**DOI:** 10.1186/s40644-024-00720-9

**Published:** 2024-06-23

**Authors:** Li Zhu, Jiali Liu, Liang Zeng, Sohun Moonindranath, Peng An, Hu Chen, Quanyong Xiang, Zhongqiu Wang

**Affiliations:** 1 Department of Radiology, Affiliated Hospital of Nanjing University of Chinese Medicine, No. 155 Hanzhong Road, Nanjing, 210029 China; 2 School of Public Health, Southeast University, No. 2 Sipai Lou, Nanjing, 210096 China; 3 Department of Chronic Non-communicable Disease Control, Jiangsu Provincial Center for Disease Control and Prevention, 172 Jiangsu Road, Nanjing, 210009 China; 4 Clinical Oncologist@ New Cancer Centre, Vacoas-Phoenix, Mauritius

**Keywords:** Preserved ratio impaired spirometry, Lung cancer, Chronic obstructive pulmonary disease, High resolution computed tomography

## Abstract

**Purpose:**

Preserved ratio impaired spirometry (PRISm) and chronic obstructive pulmonary disease (COPD) belong to lung function injury. PRISm is a precursor to COPD. We compared and evaluated the different basic information, imaging findings and survival curves of 108 lung cancer patients with different pulmonary function based on high resolution computed tomography (HRCT).

**Methods:**

This retrospective study was performed on 108 lung cancer patients who did pulmonary function test (PFT) and thoracic HRCT. The basic information was evaluated: gender, age, body mass index (BMI), smoke, smoking index (SI). The following pulmonary function findings were evaluated: forced expiratory volume in 1s (FEV_1_), forced vital capacity (FVC), FEV_1_/FVC ratio. The following computed tomography (CT) findings were evaluated: appearance (bronchiectasis, pneumonectasis, atelectasis, ground-glass opacities [GGO], interstitial inflammation, thickened bronchial wall), diameter (aortic diameter, pulmonary artery diameter, MPAD/AD ratio, inferior vena cava diameter [IVCD]), tumor (volume, classification, distribution, staging [I, II, III, IV]). Mortality rates were calculated and survival curves were estimated using the Kaplan-Meier method.

**Results:**

Compared with normal pulmonary function group, PRISm group and COPD group were predominantly male, older, smoked more, poorer lung function and had shorter survival time after diagnosis. There were more abnormal images in PRISm group and COPD group than in normal lung function group (N-C group). In PRISm group and COPD group, lung cancer was found late, and the tumor volume was larger, mainly central squamous carcinoma. But the opposite was true for the N-C group. The PRISm group and COPD group had significant poor survival probability compared with the normal lung function group.

**Conclusions:**

Considerable differences regarding basic information, pulmonary function, imaging findings and survival curves are found between normal lung function group and lung function injury group. Lung function injury (PRISm and COPD) should be taken into account in future lung cancer screening studies.

**Supplementary Information:**

The online version contains supplementary material available at 10.1186/s40644-024-00720-9.

## Background

In China, lung cancer is the main cause of cancer-associated mortality and morbidity. Some studies have shown that about 787,000 new cases of lung carcinoma were confirmed in the People’s Republic of China in 2015, with an average of over 2,100 new lung cancer diagnosed every day [1]. With the popularity of high resolution computed tomography (HRCT), the detection rate of lung cancer is rising year by year. HRCT can find lung cancer with early stage which often appears as ground-glass nodules (GGNs) [2]. According to the National Lung Screening Trial (NLST), HRCT-based lung cancer screening is receiving increasing attention [3].

Some studies demonstrated that lots of lung cancer patients have a past history of chronic obstructive pulmonary disease (COPD) [4]. COPD is characterized by chronic airway inflammation and persistent airflow restriction. Lung cancer is also the main reason of death, second only to cardiovascular diseases [5]. Symptoms of COPD range from chronic productive cough to expiratory dyspnea [6]. Previous studies have proved an association between COPD and lung cancer [7]. COPD is deemed as an important risk factor for lung cancer while lung cancer is a common complication of COPD [8]. In an epidemiological survey, adenocarcinoma is the most frequent in Stage I of Global Initiative for Chronic Obstructive Lung Disease (GOLD), while squamous carcinoma is common in Stages II and III of GOLD [9].

Recent studies have shown that E-cadherin and β-catenin were reduced in epithelial cells of COPD patients who smoke repeatedly [10]. In the airways, epithelial-mesenchymal transition (EMT) has been connected with metaplasia, gene mutation, hypertrophy, and modification of lung epithelial cells which is deemed to a important mechanism in the nosogenesis or transformation of COPD [11].

Smoking is a major factor leading to the pathogenesis and development of lung diseases [12]. The mixture of gases produced by smoking has been shown to contain about 4,500 components such as nicotine, carbon monoxide, oxidants, aldehydes, and fine particles [13]. It is well established that cigarette smoking and COPD should be deemed as crucial hazard factors for carcinoma of the lungs [14].

Many previous studies have investigated the relationship between lung cancer and COPD with varying results, but few studies have combined image representation. In this study, we summarized and analyzed the image performance and pulmonary function of 108 patients with lung cancer. We used thoracic HRCT and lung function test to understand the possible link between cigarette smoking, COPD and lung cancer, so as to provide theoretical basis for follow-up studies on the prevention and treatment of lung cancer.

## Methods

### Patients

**Fig. 1:**
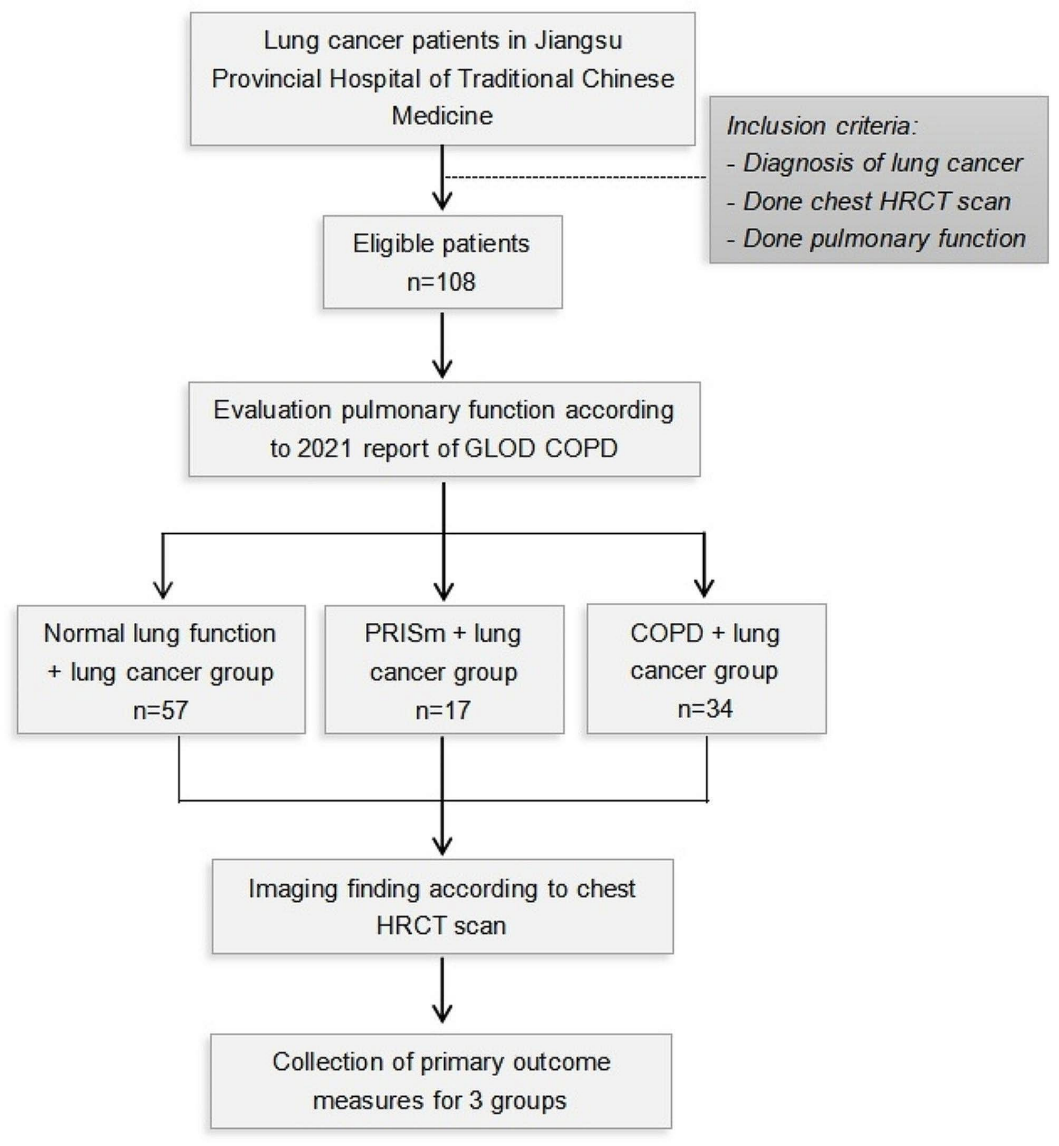
Study design of computed tomography screening for normal lung function + lung cancer group, PRISm + lung cancer group and COPD + lung cancer group in Nanjing. GLOD: Global Initiative on Chronic Obstructive Pulmonary Disease; COPD: Chronic Obstructive Pulmonary Disease; PRISm: Preserved Ratio-Impaired Spirometry; HRCT: High Resolution Computed Tomography

Medical records were retrospectively reviewed for 108 Chinese patients, between August 1, 2018, and December 30, 2020, who had been diagnosed as lung cancer by pathology and who sought medical care at the Affiliated Hospital of Nanjing University of Chinese Medicine. Eligible patients who did pulmonary function test and chest HRCT were recruited. The time intervals between pathological diagnosis, CT and PFT were not more than 5 days. Those patients were classified as having normal lung function based on FEV_1_ ≥ 80% and FEV_1_/FVC ratio ≥ 70%, COPD (GOLD stages 1–4) and Preserved ratio impaired spirometry (PRISm) (FEV_1_ < 80% and FEV_1_/FVC ratio ≥ 70%) [15]. The purpose of the smoking index (SI) is to calculate the average smoking rate in a smoker. Formula for calculation of smoking rate is [Number of cigarettes smoked per day]×[Years spent smoking] / 20 [16]. In these cases, patients must have a preliminary diagnosis of lung cancer and agree to release their medical records for retrospection. All cancer diagnosis was done by documenting pathology. The start point for survival was the first time of pathological diagnosis. The study design is shown below (Fig. 1).

### Chest HRCT scan parameter

Eligible patients received more than 64-row multi-detector CT scanners (GE revolution 256 row [US] ; Philips iCT 256 row [US] ; Philips Brilliance64 64 row [US] ; GE Optima CT680 64 row [US] ; GE Optima CT660 64 row [US] ). The tube voltage is 120 kV and the tube current is 100–200 mA. Axial images were obtained at 1.25–2.0 mm thickness with 50% overlap and reconstructed with both soft tissue and lung kernels. Coronal and sagittal multiplanar reconstructions were reconstructed and used for interpretation. All patients were scanned in supine position and suspended inspiratory terminal volume. Conventional CT scan was performed ranging from thoracic inlet to costal septal angle.

### The HRCT measurements of the blood vessel diameter

**Fig. 2:**
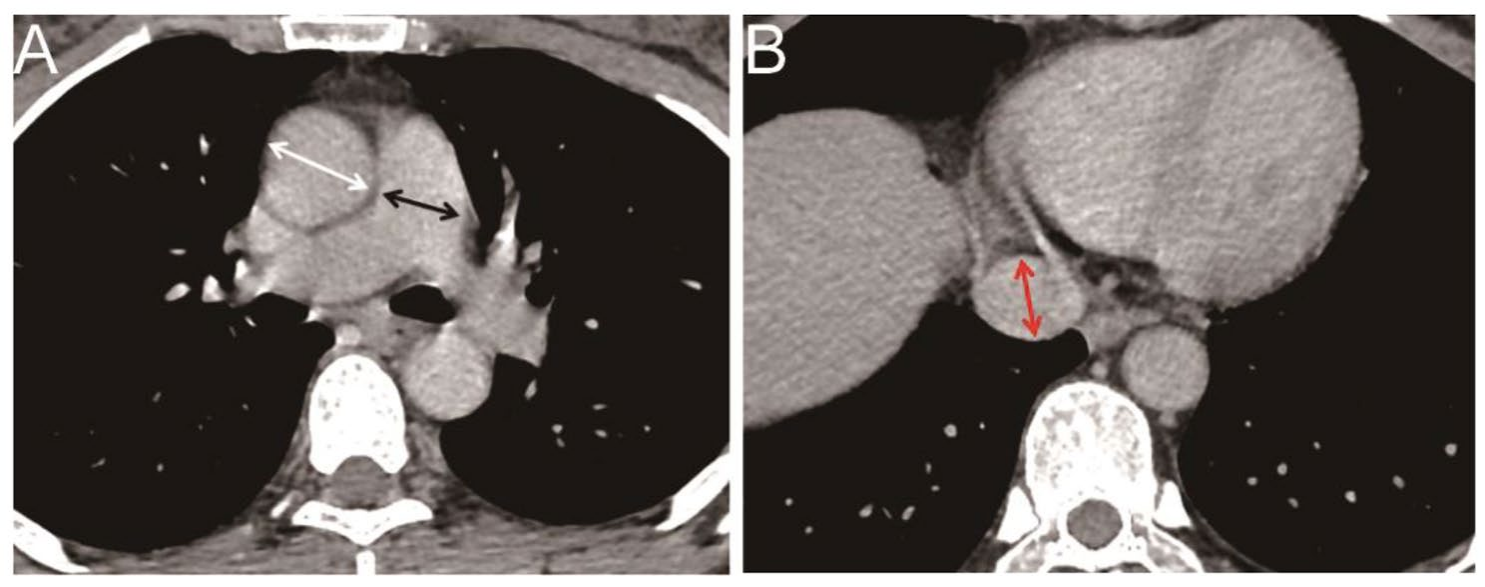
(**A**) The diameter of the main pulmonary artery at the level of its bifurcation (black line) was measured. The ascending aorta in the same image (white line) was used for evaluating the diameter of the aorta. (**B**) The short axis of the inferior vena cava (IVC) between the left atrium and hepatic vein (red line) was measured

The main pulmonary artery diameter (MPAD), the maximum dimension of the inferior vena cava (IVC), and PA–Ao ratio are indicators of right cardiac pressure (Fig. 2). At the level of the branches of pulmonary artery, the diameter of the main pulmonary artery is measured which is defined as MPAD. PA–Ao ratio is the ratio of the main pulmonary artery diameter to ascending aortic diameter at the same level of the main pulmonary artery diameter. The maximum diameter of the inferior vena cava is measured at the level of the minor axis of the inferior vena cava between the hepatic vein and the left atrium.

### Chest CT imaging abnormalities

All thoracic HRCT scans were reviewed by the agency’s digital database system (CE Workstation [GE, the United Stated] ) by two radiologists (L.ZH. and L.Z.) with 5 and 10 years of imaging experience, respectively. They both agreed to a final decision. All thoracic HRCT images were described on the basis of the Fleischner Society guidelines and peer-reviewed literature on COPD. The following CT imaging abnormalities were evaluated: appearance (bronchiectasis, pneumonectasis, atelectasis, ground-glass opacities [GGO], interstitial inflammation, thickened bronchial wall), diameter (aortic diameter, pulmonary artery diameter, PA–Ao ratio, inferior vena cava diameter [IVCD] ), tumor (volume, classification, distribution, staging [I, II, III, IV] ). The tumor volume was calculated using the formula V = 1/2×L×W×H. The unit of tumor volume is cm [3]. These are RECIST type diameter measurements. All primary tumors were single lesions.

### Statistical analysis

In this study, image measurement was achieved using a CE Workstation (GE, US). Continuous variables were represented by means with standard deviation or medians with interquartile range and ANOVA or Kruskal-Wallis H test was used for comparison between groups. Categorical variables were performed as numbers (percentages), and comparisons between groups were expressed using chi-square test or Fisher-Freeman-Halton exact test Kruskal-Wallis H test was used between ordinal categorical variables(tumor staging) of the three groups. Bonferroni correction was used to post hoc multiple comparisons(A two-side *P* < 0.05/3 was considered statistically significant).The overall survival between different groups was compared by Kaplan-Meier analysis with the log-rank test. All statistical analyses were performed using SPSS 25.0.

## Results

### Clinical characteristics of the lung cancer patients

In this study, the clinical features of the lung carcinoma patients are shown in Table 1. Patients in PRISm group (70.76 ± 6.71 year) and COPD group (67.15 ± 7.27 year) were older than N-C group (56.30 ± 14.38 year). In PRISm group and COPD group, male patients (PRISm: 13 [76.5%], COPD: 29 [85.3%] ) were more than female patients (PRISm: 4 [23.5%], COPD: 5 [14.7%] ). While the opposite results in normal lung function group (male:19 [33.3%], female: 38 [66.7%] ). BMI among the three groups was no significant difference (Table 1). By calculating the smoking index(SI), we found the patients in PRISm group (SI > 20: 52.9%) and COPD group (SI > 20: 61.8%) were smoking far more than patients in N-C group (SI > 20: 7.0%). In contrast, the pulmonary function of COPD group ( FEV_1_/FVC: 61.24 ± 8.97%) were significantly inferior to PRISm group (FEV_1_/FVC: 85.07 ± 8.67%). In addition, the pulmonary function of PRISm group (FEV_1_: 66.09 ± 10.13%, FVC: 66.22 ± 12.58%, FEV_1_/FVC: 85.07 ± 8.67%) and COPD group (FEV_1_:57.21 ± 17.54%, FVC: 69.95 ± 17.61%, FEV_1_/FVC: 61.24 ± 8.97%) were significantly worse than N-C group (FEV_1_: 99.83 ± 13.25%, FVC: 99.94 ± 14.98%, FEV_1_/FVC: 97.28 ± 11.52%).

**Table 1:** Clinical characteristics of the lung cancer patients

Characteristics	Normal lung function lung cancer group *N* = 57	PRISm lung cancer group *N* = 17	COPD lung cancer group *N* = 34	F/χ^2^	*P*-value
Age, yr	56.30 ± 14.38^#^	70.76 ± 6.71^*^	67.15 ± 7.27^*^	15.181	< 0.001
Male/Female	19/38^#^	13/4^*^	29/5^*^	26.673	< 0.001
BMI, Kg/m²	23.58 ± 4.03	22.74 ± 2.92	21.99 ± 2.92	2.159	0.121
Smoking	10(17.5%)^#^	10(58.8%)^*^	24(70.6%)^*^	27.553	< 0.001
SI > 20	4(7.0%)^#^	9(52.9%)^*^	21(61.8%)^*^	33.899	< 0.001
FEV_1,_%	99.83 ± 13.25^#^	66.09 ± 10.13^*^	57.21 ± 17.54^*^	104.975	< 0.001
FVC,%	99.94 ± 14.98^#^	66.22 ± 12.58^*^	69.95 ± 17.61^*^	54.750	< 0.001
FEV_1_/FVC,%	97.28 ± 11.52^#^	85.07 ± 8.67^*#^	61.24 ± 8.97^*^	128.654	< 0.001

F: ANOVA; χ^2^: Chi-square test; *: Compared with the normal lung function group, the results were statistically significant; #: Compared with COPD group, the results were statistically significant; BMI: Body Mass Index; SI: Smoking index; FEV_1_: Forced Expiratory Volume in 1s; FVC: Forced vital capacity

### Imaging abnormalities on HRCT

The following HRCT imaging abnormalities were evaluated: appearance (bronchiectasis, thickened bronchial wall, pneumonectasis, atelectasis, ground-glass opacities[GGO], interstitial inflammation), diameter(aortic diameter[AD], main pulmonary artery diameter[MPAD], PA–Ao ratio, inferior vena cava diameter[IVCD]) (Table 2). By statistical analysis, there were more abnormal images in PRISm group and COPD group while opposite results were found in the normal lung function group except for Ground-glass Opacities(GGO). Other imaging appearances (bronchiectasis, bronchiectasis, thickened bronchial wall, pneumonectasis, atelectasis, interstitial inflammation) in PRISm group and COPD group were more than normal lung function group. Similar results were found in diameter measurements ( IVC > 2.1 cm, AD > 3.9 cm, MPAD > 2.9 cm).

**Table 2:** Imaging abnormalities on HRCT

Imaging finding(+)	Normal lung function lung cancer group *N* = 57	PRISm lung cancer group *N* = 17	COPD lung cancer group *N* = 34	F/χ^2^	*P*-value
Bronchiectasis	14(24.6%)^#^	11(64.7%)^*^	24(70.6%)^*^	21.246	< 0.001
Thickened bronchial wall	29(50.9%)^#^	16(94.1%)^*^	34(100%)^*^	30.681	< 0.001
Pneumonectasis	6(10.5%)^#^	8(47.1%)^*^	22(64.7%)^*^	29.842	< 0.001
Atelectasis	3(5.3%)^#^	6(35.3%)^*^	10(29.4%)^*^	12.927	0.002
GGO	47(82.5%)	15(88.2%)	27(79.4%)	0.609	0.738
Interstitial inflammation	15(26.3%)^#^	12(70.6%)^*^	30(88.2%)^*^	35.330	< 0.001
IVCD > 2.1 cm	8(14.0%)	8(47.1%)^*^	10(29.4%)	8.586	0.014
AD > 3.9 cm	3(5.3%)^#^	2(11.8%)	10(29.4%)^*^	9.702^a^	0.007
MPAD > 2.9 cm	4(7.0%)	6(35.3%)^*^	5(14.7%)	7.786^a^	0.015
PA–Ao ratio	0.76 ± 0.10	0.78 ± 0.18	0.75 ± 0.10	0.324	0.724

F: ANOVA; χ^2^: Chi-square test; a:Fisher-Freeman-Halton exact test statistic; *: Compared with the normal lung function group, the results were statistically significant; #: Compared with COPD group, the results were statistically significant; GGO: Ground-glass Opacities; IVCD: Inferior Vena Cava Diameter; AD: aortic diameter; MPAD: Main Pulmonary Artery Diameter

### Imaging differences of lung cancer in three groups based on HRCT

**Fig. 3:**
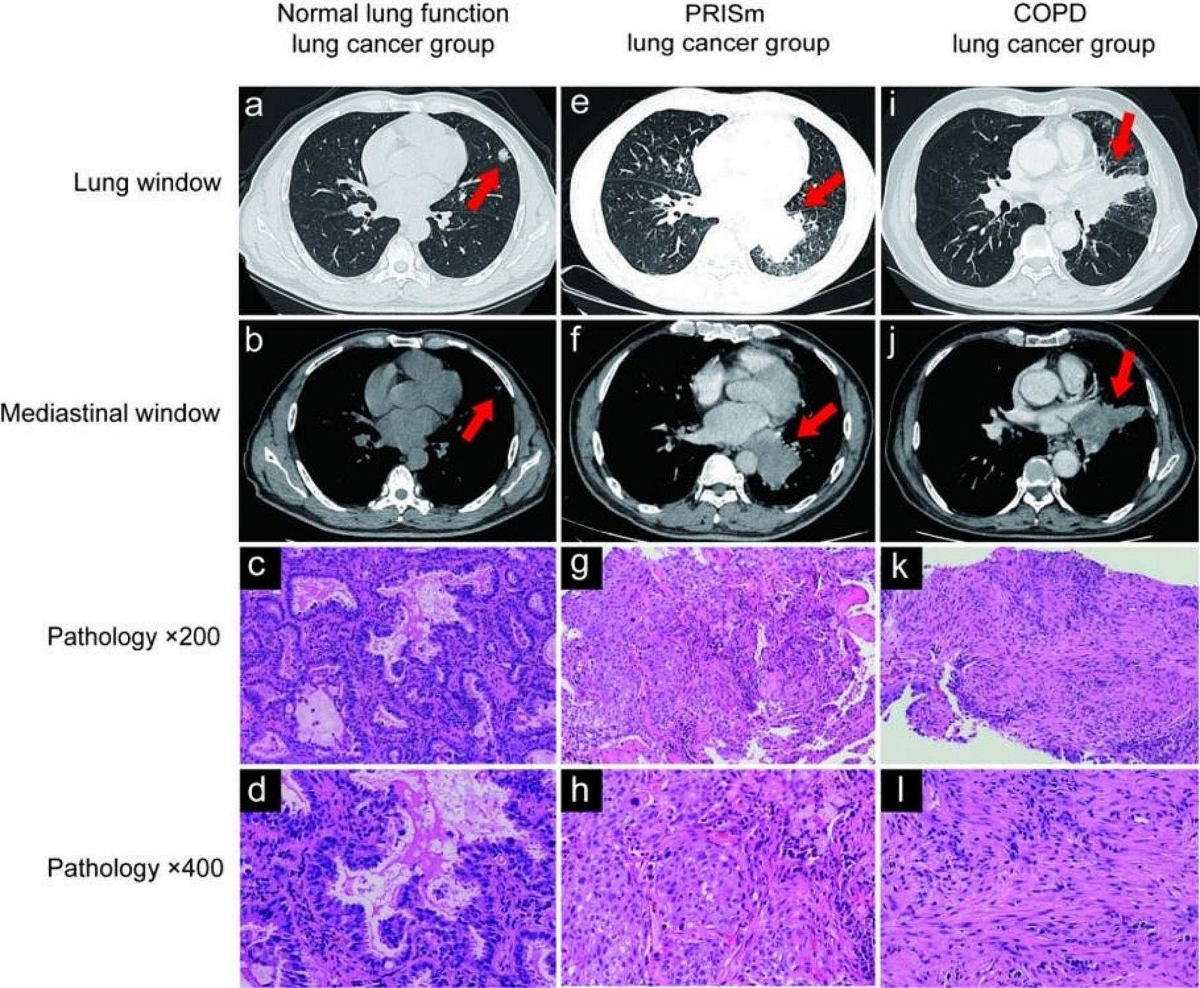
The chest HRCT scan images and pathological images in different groups of lung cancer. (**a**-**d**) Peripheral adenocarcinoma (IA2(pT1b, N0,cM0)) in a 57-year-old man with normal lung function (arrow). (**e**-**h**) Central squamous cell carcinoma (IVA(T3,N2,M1a)) in a 65-year-old man with PRISm (arrow). (**i**-**l**) Central squamous cell carcinoma (IVB(T4,N3,M1c)) in a 75-year-old man with COPD (arrow). Photomicrographs of pathology of lung cancer (original magnification, ×200; H-E stain) show in (**c**, **g**, **k**) and (original magnification, ×400; H-E stain) show in (**d**, **h**, **l**)

Among patients with PRISm and COPD, the tumor volumes were significantly larger than normal lung function group (Fig. 3; Table 3). The location of tumors in PRISm and COPD group were mainly central type ( PRISm group : 58.8%, COPD group : 52.9%), while the location of tumors in the normal lung function group were mostly peripheral type(87.7%). Pathological type of tumors of PRISm and COPD group were principally squamous carcinoma(PRISm group : 52.9%, COPD group : 44.1%), while in the N-C group, squamous carcinoma was only 1.8%. TNM staging was used for lung cancer staging, which belongs to clinical staging. Meanwhile, lung cancer staging was found later in the PRISm group(*P* < 0.001) and COPD group(*P* < 0.001) compared with N-C group and was mostly stage IV at the time of first diagnosis. In contrast, the N-C group was more likely to have stage I at first diagnosis.

**Table 3:** Imaging differences of lung cancer masses in three groups based on HRCT

Imaging finding(+)			Normal lung function lung cancer group *N* = 57	PRISm lung cancer group *N* = 17	COPD lung cancer group *N* = 34	χ^2^/H	*P*-value
Tumor	Tumor volume (cm^3^)		0.86(0.28,4.20)^#^	7.67(1.04,65.73)^*^	15.64(6.45,34.47)*	38.232	< 0.001
	Tumor type	Squamous carcinoma	1(1.8%)^#^	9(52.9%)^*^	15(44.1%)^*^	47.452^a^	< 0.001
		Adenomatous carcinoma	56(98.2%)^#^	8(47.1%)^*^	15(44.1%)^*^		
		Small-cell lung carcinoma	0	0	1(2.9%)		
		Neuroendocrine carcinoma	0	0	1(2.9%)		
		Adenosquamous carcinoma	0	0	1(2.9%)		
		Mucoepidermoid carcinoma	0	0	1(2.9%)		
	Tumor distribution	Peripheral type	50(87.7%)^#^	7(41.2%)^*^	16(47.1%)^*^	22.501	< 0.001
		Central type	7(12.3%)^#^	10(58.8%)^*^	18(52.9%)^*^	22.501	< 0.001
	Staging	I	45(78.9%)	5(29.4%)	7(20.6%)	32.670	< 0.001
		II	3(5.3%)	2(11.8%)	4(11.8%)		
		III	4(7.0%)	3(17.6%)	8(23.5%)		
		IV	5(8.8%)	7(41.2%)	15(44.1%)		

H: Kruskal-Wallis H test;χ [2]: Chi-square test; a:Fisher-Freeman-Halton exact test statistic;

*: Compared with the normal lung function group, the results were statistically significant; #: Compared with COPD group, the results were statistically significant

### Survival rate of lung cancer patients with different pulmonary function

**Fig. 4:**
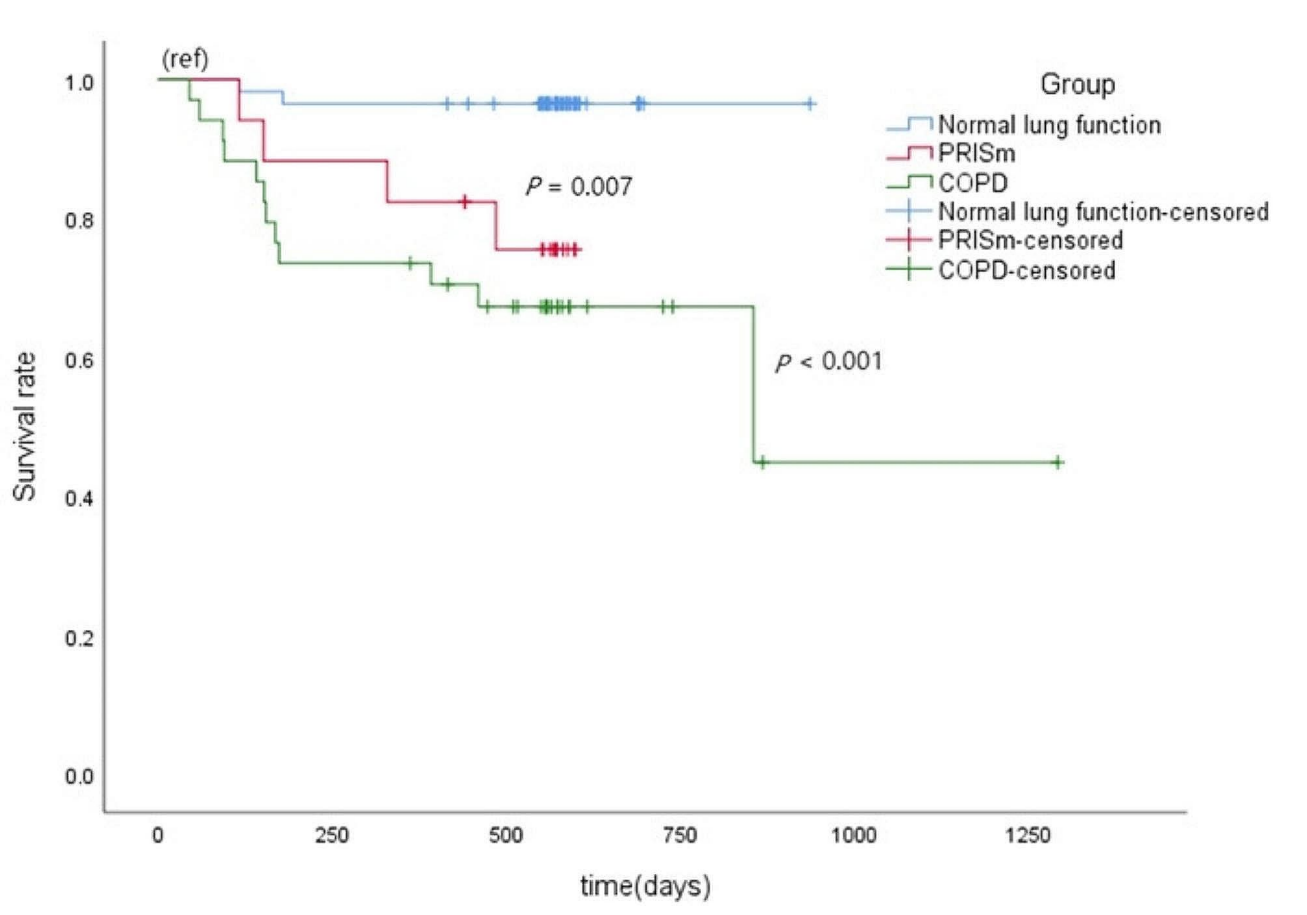
Kaplan-Meier survival analysis of survival-associated different pulmonary function represented as normal lung function, PRISm vs. COPD in lung cancer. Abbreviations: PRISm: Preserved Ratio-Impaired Spirometry; COPD: Chronic Obstructive Pulmonary Disease

Among 108 lung cancer patients, the PRISm group(*P* = 0.007) and COPD group(*P*<0.001) had significant poor survival rate compared with the normal lung function group. (Fig. 4). Survival probability at 365 days of normal lung function group, PRISm group and COPD group were 96%, 75% and 65% respectively.

## Discussion

This is a retrospective analysis of 108 patients, who did HRCT and lung function tests, confirmed with lung cancer by pathology. The main findings of this study are: (1) Lung cancer patients with impaired lung function (PRISm or COPD) are more common in men. (2) Patients with impaired lung function are older than normal lung function patients. (3) Smoking is more familiar in people with impaired lung function. (4) The indexes of lung function (FEV_1_, FVC, FEV_1_/FVC) were decreased in PRISm group and COPD group. (5) Except for Ground-glass Opacities(GGO), other imaging appearances (bronchiectasis, bronchiectasis, thickened bronchial wall, pneumonectasis, atelectasis, interstitial inflammation) in PRISm group and COPD group were more than normal lung function group. (6) The numbers of patients in PRISm group with diameter measurements (IVCD > 2.1 cm, MPAD > 2.9 cm) were more than N-C group. The numbers of patients in COPD group with diameter measurements (AD > 3.9 cm) were more than N-C group. (7) In PRISm group and COPD group, lung cancer was found late, and the tumor volume was larger, mainly central squamous carcinoma. But the N-C group had the opposite results.

Despite recent advances, lung cancer is currently the main cause of cancer-associated mortality and the most common cancer all over the world [17]. The risk factors for lung cancer include tobacco consumption, airway lesions, genetic predisposition, air pollution and others [18]. Besides age (> 60 years), smoking status has been shown to have the greatest affect on the probability of developing lung carcinoma [19]. Lung carcinoma is one of the principal causes of hospitalization and death in patients with COPD [20]. Recent analysis of emphysema and airway obstruction in lung carcinoma screening group increased our understanding of COPD and lung cancer risk [21]. Spirometry was confirmed to play a significant role in COPD early diagnosis and screening [22]. On the basis of GOLD, COPD was confirmed in case of FEV_1_ to FVC ratio is less than 0.7, which means the patient breathes out below 70% of the air from lung in one second [23]. However one category of patients has been overlooked. Patients with preserved ratio impaired spirometry (PRISm) also has a low FEV_1_, exacerbations and symptoms. However, the forced vital capacity of those patients is also low, leading to a FEV_1_ to FVC ratio greater than 0.7. Some studies show that PRISm and COPD belong to lung function injury and PRISm is a precursor to COPD [24]. Therefore, we divided 108 lung cancer patients into three groups which respectively were lung cancer coexisting with normal lung function group, lung cancer coexisting with PRISm group, lung cancer coexisting with COPD group according to lung function in this study.

In the early diagnosis of lung carcinoma, HRCT plays an important role. Detection and screening of early lung cancer is very important to improve survival rates [25]. Imaging abnormalities and measures of chest CT imaging and pulmonary function measurement were included in this study in which we found that in contrast with N-C group, PRISm group and COPD group were predominantly male, older, smoked more, poorer lung function and had shorter survival time after diagnosis. There were more abnormal images in PRISm group and COPD group than in N-C group. In PRISm group and COPD group, lung cancer was found late, larger, and mainly central squamous carcinoma. But the N-C group had the opposite findings. We found that lung cancer in normal lung function group was discovered early, smaller, and mainly adenocarcinoma. Meanwhile the PRISm group and COPD group had significant poor survival rate compared with the normal lung function group. In addition, the survival rate of PRISm group was lower than that of COPD group. This discrepancy may be due to differences in smoking, air quality, co-morbid disease and so on. The N-C group often had stage I at first diagnosis, therefore had a higher rate of surgical resection and longer survival time. However, lung cancer was found late in the PRISm group and COPD group and was mostly stage IV at the time of first diagnosis. Therefore, patients with impaired lung function (PRISm and COPD) had lower surgical resection rate and shorter survival time.

Several limitations of our study should also be noted. First, it was a single-center retrospective study and thus the number of patients enrolled was limited. This raises a difficulty when overall survival in subgroups of lung cancer is compared and limits conclusions about differences between lung cancer groups. Second, the pulmonary function examination rate of lung cancer patients is low in clinical departments, resulting in the restriction of cohort sizes. Both of these limitations may be resolved by further analysis with larger cohort to acquire definite conclusions.

## Conclusion

Considerable differences regarding basic information, pulmonary function, imaging findings and survival curves are found between normal lung function group and lung function injury group which is discovered by our research team. Lung function injury (PRISm and COPD) should be taken into account in future lung cancer screening studies.

### Electronic supplementary material

Below is the link to the electronic supplementary material.

Supplementary Material 1

## Data Availability

The datasets used and/or analyzed during the current study are available from the corresponding author on reasonable request.

## Abbreviations

PRISm: Preserved ratio impaired spirometry
COPD: Chronic obstructive pulmonary disease
HRCT: High resolution computed tomography
CT: Computed tomography
N-C group: Normal lung function group
GGNs: Ground-glass nodules
NLST: National Lung Screening Trial
GOLD: Global Initiative for Chronic Obstructive Lung Disease
EMT: Epithelial-mesenchymal transition
BMI: Body Mass Index
SI: Smoking index
FEV_1_: Forced Expiratory Volume in 1s
FVC: Forced vital capacity
GGO: Ground-glass Opacities
IVCD: Inferior Vena Cava Diameter
AD: Aortic diameter
MPAD: Main Pulmonary Artery Diameter
