# Supplementary material for: Thoracic high resolution computed tomography evaluation of imaging abnormalities of 108 lung cancer patients with different pulmonary function
Source: Cancer Imaging. 2024 Jun 23;24:78. doi: 10.1186/s40644-024-00720-9 (PMC11194896; doi:10.1186/s40644-024-00720-9)
Supplement: Supplementary file 1 — Supplementary Material 1 [file 40644_2024_720_MOESM1_ESM.docx]

**Manuscript ID number:**

CAIG-D-22-00203

**Title of paper:**

Thoracic High Resolution Computed Tomography Evaluation of Imaging Abnormalities of 108 Lung Cancer Patients With Different Pulmonary Function

Dear Editors,

We are writing to re-submit the revised manuscript entitled “Thoracic high resolution computed tomography evaluation of imaging abnormalities of 108 lung cancer patients with different pulmonary function” (CAIG-D-22-00203). I would like to take this opportunity to thank the reviewers to provide invaluable comments for the improvement of our manuscript. We have carefully studied the comments of the reviewers and made necessary changes in the revised manuscript. We hope that we have addressed all of the questions satisfactorily and the manuscript at current version can be accepted in your journal. I am looking forward to receiving your favor reply.

Thank you and best regards.

Yours sincerely,

Corresponding Author:

Professor Quanyong Xiang

School of Public Health, Southeast University; Department of Chronic Non-communicable Disease Control, Jiangsu Provincial Center for Disease Control and Prevention.

Nanjing, China.

Tel: 86-25-83759469 (office); 86-18118996918(mobile).

E-mail:[quanyongxiang@vip.sina.com](mailto:quanyongxiang@vip.sina.com)

[Joint corresponding author](javascript:;):

Professor Zhongqiu Wang

Department of Radiology, Affiliated Hospital of Nanjing University of Chinese Medicine

Nanjing, China

Tel: 86-25-85811065(office); 86-13905162963(mobile).

E-mail:[zhongqiuwang0815@163.com](mailto:zhongqiuwang0815@163.com)

Q1 : It would improve this manuscript if the authors could clarify Figure 1, which shows a point of inclusion criteria, but then implies CT and PFT being done after selection? Or was that data all retrospective?

**Author’s Response :** Yes, that data was all retrospective. For details, please see line 89 on page 3 in manuscript.

Q2 : What were the time intervals between Diagnosis, CT and PFT?

**Author’s Response :** Yes, the time intervals between pathological diagnosis, CT and PFT were not more than 5 days. For details, please see line 92-93 on page 3 in manuscript.

Q3 : Was cancer diagnosis all done by documenting pathology (FNA, resection, etc)?

**Author’s Response :** Yes. For details, please see line 100 on page 3 in manuscript.

Q4 : Regarding Figure 4 Survival, what was the start point for Survival, from presentation, or from path report date?

**Author’s Response:** Yes, the start point for survival was the first time of pathological diagnosis. For details, please see line 100-101 on page 3 in manuscript.

Q5 : Some discussion should be given as to whether the COPD and PRISm groups are distinguishable by the studies - although Survival may be different?

**Author’s Response :** Yes, a discussion of COPD and PRISm has been added to the manuscript, including a discussion of survival. For details, please see line 277-279 on page 12 in manuscript.

Q6 : What Staging system was used? TNM or AJCC are well known, other? And were these clinically or surgically staged?

**Author’s Response :** Yes, TNM staging was used for lung cancer staging, which belongs to clinical staging. For details, please see line 207-208 on page 8 in manuscript.

Q7 : Survival obviously will be impacted by treatment. Ideally, one should know whether resection, chemo, targeted, immunotherapy, etc. played a role, but those questions would bring up questions of EGFR and many other tumor mutations, beyond the scope of this paper, but worth comment.

**Author’s Response :** Yes, the N-C group often had stage I at first diagnosis, therefore had a higher rate of surgical resection and longer survival time. However, lung cancer was found late in the PRISm group and COPD group and was mostly stage IV at the time of first diagnosis. Therefore, patients with impaired lung function (PRISm and COPD) had lower surgical resection rate and shorter survival time. For details, please see line 279-283 on page 12 in manuscript.

Q8 : Regarding Table 3, and methods, how was Tumor Volume calculated? And what units (mL or cc?).

**Author’s Response :** Yes, the tumor volume was calculated using the formula V=1/2×L×W×H. The unit of tumor volume is cm^3^. For details, please see line 144-145 on page 5 in manuscript. In addition,we found that tumor volume did not follow a normal distribution, so we changed the mean ± standard deviation into the median (interquartile range) and re-selected the correct statistical method (Kruskal-Wallis H test).However the results obtained by these two statistical methods were similar. For details, please see table 3 on page 9 (Tumor volume)in manuscript.

Q9 : Are these RECIST-type diameter measurements? Single lesions or multiple?

**Author’s Response :** Yes, these are RECIST type diameter measurements. All primary tumors were single lesions. For details, please see line 145-146 on page 5 in manuscript.

Q10 : And Table 3 needs clarification of the Tumor type % breakdown, we don't see how 100% would be reached.

**Author’s Response :** Yes, the information in the Table 3 has been supplemented and completed. For details, please see line 219 on page 9 in manuscript.

**In addition, we made the following modifications in this paper.**

Modification in Table 2:

Due to our mistake, we misjudged the conditions of application of chi-square test, which led to the incorrect use of Fisher-Freeman-Halton exact test. We used Fisher-Freeman-Halton exact test for the previous statistics. But after a careful review of the relevant literature, we found that the chi-square test can be used. The reasons are as follows:

In the contingency table of R*C, the application condition of Chi-square test requires that the theoretical frequency should not be too small (In 1954, Cochran defined the theoretical frequency too small as: More than 1/5 cells have a theoretical frequency less than 5 or 1 cell has a theoretical frequency less than 1). If the above conditions are not satisfied, the Fisher-Freeman-Halton exact test can be used. However, in our modified data, we found that the theoretical frequency of only one cell (16.7%) was less than 5, and the theoretical frequency was all greater than 1, which met the condition of Chi-square test. Therefore, we choosed to use the statistical results of chi-square test. Although two statistical methods were used before and after the modification, the results obtained by these two statistical methods were similar. For details, please see table 2 on page 7 in manuscript.

Modification in Statistical analysis:

In order to facilitate the reader's understanding, we added some descriptions of statistical methods.For details. Please see the statistical analysis part of the method on page 6.
